# Sketches from the Case-Book, to Illustrate the Influence of the Mind on the Body; with the Treatment of Some of the More Important Brain and Nervous Disturbances Which Arise from This Influence

**Published:** 1833-07

**Authors:** 


### BIBLIOGRAPHICAL NOTICE.

*Sketches from the Case-book, to illustrate the Influence of the Mind on the Body; with the Treatment of some of the more important Brain and Nervous Disturbances which arise from this Influence.* By R. Fletcher, Esq., Surgeon to the Glocester General Hospital, &c.—8vo. pp. 391. Longman, London.

Unless we are mistaken, there are many who suppose that a reviewer feels a secret satisfaction in "cutting up" the works that fall under his notice; but we can declare most conscientiously for ourselves, that there is no duty more irksome to us, none more painful to our feelings, than that of commenting with severity upon the labours of others. Disagreeable, however, as the duty is, it is imperative upon us, as honest journalists, and it must be performed.

We derived so much pleasure and instruction from the perusal of Mr. Fletcher's former works, that we approached the consideration of the present with our minds made up in his favour, and with a feeling amounting almost to a conviction that we should again have to offer him our meed of approbation, and afford our readers the opportunity, by a long analysis, of judging for themselves of the justice of our critical decision. We cannot, however, either bestow the smallest portion of praise upon the present work, or occupy our pages by any lengthened extracts from it.

The title naturally led us to look for some practical information; and we had previous reasons for believing that Mr. Fletcher's good taste would ensure us a proper seriousness of diction, and an appropriate gravity of style, in the discussion of a serious subject. But of practical information we can find very few traces; and nothing can possibly be more extravagantly trifling than the style in which the book is written. One or two examples will suffice to shew that we have "reason for our saying."

In commenting upon the regulation of diet, at page 194, Mr. F. tells us, that "fat, pure fat, and melted butter, (according to books, the greatest enemy a weak stomach can encounter,) will sometimes, most disrespectfully and spitefully to the learned authors of the said books, sit scornfully triumphant in the legitimate habitation of water-gruel!" "Mutton-chops, so famous in the treatment of the dyspeptic stomach, must now hide their stale insignificance in the presence of sliced fat bacon, which, with conscious pride of its superiority, is found upon every breakfast-table, proudly curled up in bold and impudent convexity, and ready for a start over the now neglected form and fallen fortune of the mutton-chop!" This is at least very original; for certainly nobody ever before heard of the "conscious pride" of a rasher of bacon, or ever suspected that it could be "ready for a start" over a mutton-chop.

Mr. Fletcher's grand mode of relieving mental affliction appears to us as whimsical as the terms in which he conveys his opinions. From whatever source the mental malady proceeds, the patient is to compose himself by *composition.* Is he melancholy, or three parts mad, he is to be cured by writing about any thing, or nothing. No matter whether he has taste or talent for the occupation, he is to write, and write again, and ere long he will himself be right.

We shut the book, with deep regret that Mr. Fletcher ever wrote it; but not without hopes that he will again come before the profession, and again claim the approbation of his brethren, by some performance which will not diminish the reputation he fairly acquired by the publication of his "Medico-chirurgical Notes and Illustrations," of which we thought so favorably, that we not only gave a full analysis of it, but extracted several of the cases for our Collectanea department.

